# Unravelling the Thermal Decomposition Parameters for The Synthesis of Anisotropic Iron Oxide Nanoparticles

**DOI:** 10.3390/nano8110881

**Published:** 2018-10-29

**Authors:** Geoffrey Cotin, Céline Kiefer, Francis Perton, Dris Ihiawakrim, Cristina Blanco-Andujar, Simona Moldovan, Christophe Lefevre, Ovidiu Ersen, Benoit Pichon, Damien Mertz, Sylvie Bégin-Colin

**Affiliations:** 1 Institut de Physique et Chimie des Matériaux de Strasbourg, UMR 7504, University of Strasbourg, CNRS, F-67034 Strasbourg, France; geoffrey.cotin@ipcms.unistra.fr (G.C.); celine.kiefer@ipcms.unistra.fr (C.K.); francis.perton@ipcms.unistra.fr (F.P.); dris.ihiawakrim@ipcms.unistra.fr (D.I.); cristina.blancoandujar.09@ucl.ac.uk (C.B.-A.); simona.moldovan@ipcms.unistra.fr (S.M.); Christophe.lefevre@ipcms.unistra.fr (C.L.); ovidiu.ersen@ipcms.unistra.fr (O.E.); benoit.pichon@ipcms.unistra.fr (B.P.); damien.mertz@ipcms.unistra.fr (D.M.); 2 Labex CSC, Fundation IcFRC/University of Strasbourg, 8 allée Gaspard Monge BP 70028 F, 67083 Strasbourg CEDEX, France

**Keywords:** iron oxide nanoparticles, synthesis, shape control, thermal decomposition, precursor

## Abstract

Iron oxide nanoparticles are widely used as a contrast agent in magnetic resonance imaging (MRI), and may be used as therapeutic agent for magnetic hyperthermia if they display in particular high magnetic anisotropy. Considering the effect of nanoparticles shape on anisotropy, a reproducible shape control of nanoparticles is a current synthesis challenge. By investigating reaction parameters, such as the iron precursor structure, its water content, but also the amount of the surfactant (sodium oleate) reported to control the shape, iron oxide nanoparticles with different shape and composition were obtained, in particular, iron oxide nanoplates. The effect of the surfactant coming from precursor was taking into account by using *in house* iron stearates bearing either two or three stearate chains and the negative effect of water on shape was confirmed by considering these precursors after their dehydration. Iron stearates with three chains in presence of a ratio sodium oleate/oleic acid 1:1 led mainly to nanocubes presenting a core-shell Fe_1−x_O@Fe_3−x_O_4_ composition. Nanocubes with straight faces were only obtained with dehydrated precursors. Meanwhile, iron stearates with two chains led preferentially to the formation of nanoplates with a ratio sodium oleate/oleic acid 4:1. The rarely reported flat shape of the plates was confirmed with 3D transmission electronic microscopy (TEM) tomography. The investigation of the synthesis mechanisms confirmed the major role of chelating ligand and of the heating rate to drive the cubic shape of nanoparticles and showed that the nanoplate formation would depend mainly on the nucleation step and possibly on the presence of a given ratio of oleic acid and chelating ligand (oleate and/or stearate).

## 1. Introduction

In the recent years, synthesis of magnetic nanomaterials and especially nanoparticles (NPs) have been investigated for various applications, due to their unique magnetic properties [1,2,3,4,5,6,7,8]. The shape control of iron oxide nanoparticles (IONPs) is currently considered as promising to enhance the effective anisotropy and/or surface energy of IONPs, which are required for given applications. Indeed, modifying the shape of the NPs will have different impacts. Specific shapes will be enclosed by different crystallographic planes that present different surface energies. NPs with specific surface energy is rather important as catalytic applications will look for high energy surfaces [9,10] and they also find applications as biosensors [11]. However, anisotropic shapes interest does not only rely on the NPs surface energy. Modifying the morphology will also have an impact on the magnetic properties by adding shape anisotropy to the NPs effective anisotropy [11,12,13,14,15,16]. Therefore, IONPs with anisotropic shape have been investigated for biomedical applications and especially for magnetic hyperthermia and magnetic resonance imaging (MRI) applications [17,18,19]. 

Among the most interesting shapes of IONPs reported, nanocubes and octopod shaped NPs stand out. Nanocubes of 19 nm have been shown to be very good heating agents for therapy by magnetic hyperthermia [20]. It has been shown that the cubic morphology boosted the magnetic properties of IONP [20,21,22,23]. Furthermore, NPs with faceted shapes exhibited very high contrast enhancement properties promising for imaging by MRI [14,15,24]. Octopod IONPs were demonstrated to be high-performant T_2_ contrast agents for MRI [15].

However, the reproductible control of the NPs shape is not an easy task and remains a challenge. Among current synthesis methods, the thermal decomposition (TD) one is the most developed to tune the size and shape of IONPs. It involves the thermal decomposition of an iron precursor in presence of ligands in an organic solvent [25,26]. Commonly, iron precursors, such as acetylacetonates [23,27,28], acetates [23], or oleates [25,26,27,29], have been decomposed in organic solvents containing surfactants. The strength of this method comes from the possibility to easily separate the NPs nucleation and growth steps following the theory of nucleation-growth proposed by Lamer [30]. This separation is the key parameter as it allows, by playing on synthesis parameters, to tune the size and shape of NPs. Most of the research on the TD synthesis pointed on the major role of the surfactants or ligands on the NPs features. The ligands role is, at first, to coat in situ the NPs preventing aggregation, but it has been demonstrated that ligands can delay (carboxylic acid) or accelerate (amine) the decomposition of the iron precursor [31,32], which will impact the nucleation process. Furthermore, ligands present in the reactant media will direct the growth of the nuclei and may lead towards anisotropic shapes as widely reported [15,18,19,23,24,28,33,34,35,36,37,38].

Kovalenko et al. [28] reported at first the synthesis of nanocubes by decomposing an iron oleate precursor using a sodium oleate (NaOl) salt along with oleic acid (OA) as ligands. More recently, nanocubes have been synthesized by thermal decomposition by adding dibenzylether (DBE) as solvent in presence of non-chelating ligands, such as oleic acid [21,39]. Guardia et al. [21] demonstrated that the shape control was induced by the product of decomposition of DBE at high temperature. While most of the reports emphasize the role of the ligands, Kim [39] underlined also the role of a kinetically controlled growth under high monomer concentration. Currently, most published results on the synthesis of nanocubes are based on the Kovalenko’s method using different combination of ligands (NaOl/OA [22,38,40,41,42]–OA/Oleylamine [43]—OA/4-Bisphenyl carboxilic acid [39,44]) and iron precursors are mainly iron oleate or iron acetylacetonate. We recently demonstrated that the water content was an important parameter to control the cubic shape as it induces the formation of NaOl inverse micelles affecting thus the amount of NaOl available to drive a specific growth [45]. Few papers reported the synthesis of iron oxide nanoplates by the thermal decomposition methods. Zhou et al. [24] succeeded in the synthesis of cubes and plates among other shapes of IONPs by varying the ratio FeOl/NaOl and the reaction temperature. Within the many protocols published for the synthesis of anisotropic shapes, every protocol differed from the other involving different heating rates, different ligands, and different concentration, as illustrated in Table 1, and no defined trend came out from the literature.

Yet, the ligands used in the synthesis and the heating rates appeared to be the main parameters for the shape control. However, the synthesis mechanism of anisotropic shape is not yet clearly understood. 

In our group, core-shell Fe_1−x_O@Fe_3−x_O_4_ nanocubes [48,49] were previously synthesized by adapting the Kovalenko’s method [28] (sodium oleate and oleic acid ratio 3.6:1) using an *in house* iron oleate precursor. Nevertheless, the shape quality of nanocubes was highly dependent on the iron oleate batch and several iron oleate batches were often synthesized without succeeding in obtaining homogeneous nanocubes [45]. Replacing iron oleate by a commercial iron stearate led to shape heterogeneity. By synthesizing *in house* iron stearates bearing either two (FeSt_2_) or three (FeSt_3_) stearate chains and either hydrated or dehydrated, we showed that the hydration rate of iron stearates was decisive [45]. Nanocubes with straight faces presenting a core-shell composition Fe_1−x_O@Fe_3−x_O_4_ were only obtained with dehydrated FeSt_3_. Dehydrated FeSt_2_ led to rounded cubes showing an effect of the nature of the precursor that was explained by different thermal stability of stearates, which influence the kinetics of the monomer generation thus the nucleation.

One may notice that very few papers dealt with the influence of the iron precursor structure on the formation of anisotropic shape by the thermal decomposition method. The pioneer work of Bronstein et al. [29,50] pointed at the role of the precursor structure, which would be affected by washing solvents and that was supported by Buck et al. who reported on the role of the precursor during the study of cobalt oleate (CoOl_2_) [51]. 

Considering that the synthesis of anisotropic shaped NPs is very important, due to their enhanced properties, the understanding of their synthesis mechanisms is still challenging. In that context and based on our previous investigations [45,48], we propose to investigate the effect of the structure of the precursor, which has been poorly studied up to now, and of the amount of sodium oleate on the nanoparticle shape. That’s why in this work, four precursors, hydrated and dehydrated FeSt_2_ and FeSt_3_, have been decomposed in anisotropic conditions from an adapted Kovalenko’s method [48]. Some important parameters have been thus investigated to better understand the mechanism addressing the shape of NPs: The influence of the structure (and hydration degree) of iron stearate, the ratio sodium oleate/oleic acid (NaOl/OA) and the heating rate. Then, NPs with cubic, octopod and nanoplate shapes have been structurally characterized.

## 2. Experimental Details

### 2.1. Synthesis Methods

**Synthesis of iron stearate precursors**. Iron stearate (II) and (III) were prepared by precipitation of sodium stearate (98.8% TCI, TCI Europe N.V., Zwijndrecht, Belgium), and ferrous chloride (99%, Acros Organic, Thermo Fischer Scientific, Geel, Belgium) or ferric chloride (99%, Sigma, Sigma Aldrich, Lyon, France) salts in an aqueous solution as previously reported [46]. Briefly, sodium stearate (9.8 g, 32 mmol) was transferred into a two necked round bottomed flask (RBF) and solubilized in distilled H_2_O (H_2_O, 80 mL). The solution is heated to reflux and stirred for 30 min until complete dissolution of the stearate. Separately, FeCl_2_·4H_2_O (3.16 g, 16 mmol) or FeCl_3_·6H_2_O (2.88 g, 10.6 mmol) was dissolved in H_2_O (40 mL) and added onto the sodium stearate solution under vigorous stirring. A light orange precipitate is formed immediately. The solution is kept under stirring at this temperature for 15 min. Thereafter the solution is allowed to cool down to room temperature (RT). The obtained precipitate is washed once by centrifugation (hot H_2_O, 14,000 rpm, 10 min). The product is then filtrated with a büchner funnel and oven dried at 65 °C for 24 h. Dehydration of the precursor is performed in an oven at 140 °C for 48 h. 

**NPs Synthesis** is adapted from the work of Pichon et al. [48]. 2.32 mmol of iron stearate is mixed with 3 mmol of ligands (oleic acid (OA, 99%, Alfa Aesar, Thermo Fisher GmbH, Karlsruhe, Germany) and sodium oleate (NaOl, 97%, TCI) at different ratios) in 15 mL octadecene (OD, 90%, Alfa Aesar). The mixture is stirred and heated at 120 °C for 60 min without reflux condenser in order to dissolve the reactants and remove the water residues. The cooler is then connected to the flask and the solution is heated up to 200 °C for 10 min with a heating rate of 5 °C/min. The solution is then heated up to 315 °C with a heating rate of 1 °C/min and refluxed for 60 min under air. After cooling to RT, a black and viscous suspension is obtained that is solubilized in 10 mL of chloroform. The NPs are then precipitated by the addition of an excess of acetone the first time and washed three times with chloroform and acetone at a ratio of 1:4 at 14,000 rpm for 5 min by centrifugation. The NPs can finally be suspended in 50 mL of tetrahydrofurane (THF).

**Synthesis of optimized cubes** id adapted from [45,48]. Iron oxide nanocubes (NC) were synthesized from iron stearate (III). Iron (III) stearate (2.72 g, 3 mmol) was mixed with OA (0.45 g, 1.5 mmol) and NaOl (0.45 g, 1.5 mmol) in 15 ml 1-eicosene (EC, 80%, Sigma Aldrich, Lyon, France) in a two neck- RBF. The mixture is heated to 120 °C under stirring and kept at this temperature for 30 min without reflux condenser in order to dissolve the reactants and remove the water residues. The condenser was then connected to the flask and the solution heated to boiling temperature (≈343 °C, 15 °C/min). The solution was kept at reflux for 90 min under air. After cooling to RT, a black gel was obtained. The NPs were washed as previously described. 

**Synthesis of octopods** Iron oxide Nano-octopods (NO) were synthesized from commercial iron stearate (III) (>60%, TCI). Iron (III) stearate (0.735 g, 0.9 mmol) was mixed with OA (1.02 g, 3.6 mmol) in 20 mL dibenzylether (DBE, 99%, Acros Organic) in a two neck-RBF. The mixture is heated at 120 °C under stirring and kept at this temperature for 60 min without reflux condenser in order to dissolve the reactants and remove the water residues. The condenser was then connected to the flask and the solution heated to 250 °C (5 °C/min) and kept at this temperature for 60 min. The solution was then brought to 320 °C (10 °C/min) and kept at reflux temperature for 60 min under air. After cooling to RT, a black solution was obtained. The NPs were washed as previously described. 

### 2.2. Characterization Methods

NPs were characterized by transmission electron microscopy (TEM, JEOL, Tokyo, Japan) with a JEOL 2100 microscope operating at 200 kV (point resolution 0.18 nm). 

The mean size of NPs and their size distribution were calculated from the size measurements of more than 300 nanoparticles using ImageJ software (NIH, Bethesda, MD, USA). The error presented correspond to the standard deviation of the mean diameter determined from all these size measurements.

The X-ray diffraction (XRD, Bruker, Billerica, MA, USA) pattern was recorded at room temperature with a Bruker D8 Advance diffractometer equipped with a monochromatic copper radiation source (Kα = 0.154056 nm) and a Lynx-Eye detector in the 27–65° (2θ) range with a scan step of 0.03°. High purity silicon powder (a = 0.543082 nm) was systematically used as an internal standard. Profile matching refinements were performed through the Fullprof program [52] using Le Bail’s method [53] with the modified Thompson-Cox-Hasting pseudo-Voigt profile function. 

Standard Infrared spectra were recorded between 4000 and 400 cm^−1^ with a Fourier transform infrared (FTIR) spectrometer, Spectrum 100 from Perkin Elmer (Perkin Elmer, Waltham, MA, USA). Samples were gently ground and diluted in non-absorbent KBr matrixes.

## 3. Results and Discussion

### 3.1. Influence of the Structure of Precursors and Sodium Oleate Ligand

FeSt_2_ and FeSt_3_ have been synthesized by a coprecipitation method using FeCl_2_·4H_2_O and FeCl_3_·6H_2_O respectively and these stearates have been heat-treated at 140 °C for 48 h to remove most of the crystallized water molecules leading to partially dehydrated stearates (named here FeSt_3,d_ and FeSt_2,d_) [45]. All stearates are composed of Fe^3+^, due to the oxidative synthesis condition (except FeSt_2_ that present a minor Fe^2+^ contribution), but their structure differed due to the different amount of stearate ligand and different Fe III-carboxylate coordinations (a mixture of bridging and chelating coordinations). No sodium or chloride from reactants are detected in the final product meaning that water molecules stabilize the Fe^3+^ (or Fe^2+^) center along the carboxylates (to be published results). We showed that the thermal stability of the different iron stearates is highly dependent on their structure, as well as on their hydration degree [45]. 

Spherical and cubic iron oxide NPs have been then synthesized by thermal decomposition of these precursors in presence of a given alount of surfactants in a high boiling solvent. These precursors have been decomposed using two standard protocols: One for the synthesis of 10 nm sized spherical nanoparticles (with oleic acid as surfactant and in dioctyl ether (B_p_ = 290 °C)) and the other one for the synthesis of nanocubes (with a mixture of oleic acid and sodium oleate as surfactant and in octadecene (B_p_ = 315 °C)) [45]. The size of both shaped NPs was found to be dependent on the water content, as well as the cubic morphology [45]. We suggested that the presence of water makes that oleates, used to drive the cubic shape, form micelles instead of stabilizing specific faces of nuclei. That would explain that the cubic morphology is observed only when the water content is low. The nanosphere and nanocube sizes were related to the iron stearate decomposition kinetics: FeSt_2_ and FeSt_2,d_ decomposed in larger amount at lower temperature than FeSt_3_ and thus favor the nucleation instead of the growth step, leading finally to smaller sized NPs [45]. 

**Figure 1:**
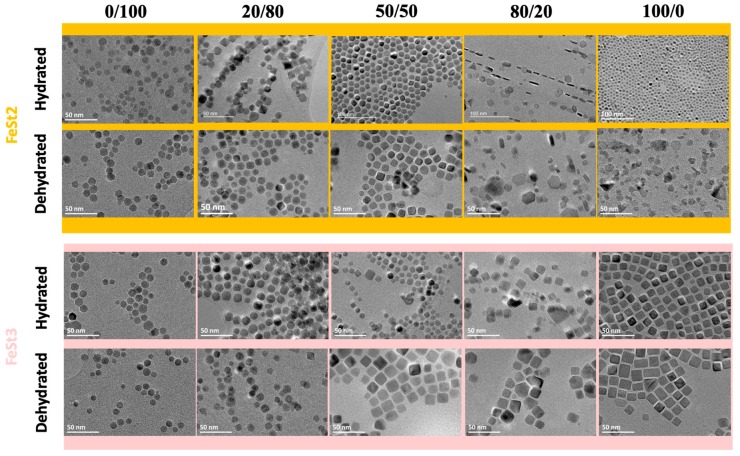
Influence of the ratio NaOl/OA on the shape of the nanoparticles (NPs) depending on the precursor structure and hydration degree.

Considering the important role of water content, which would affect the amount of sodium oleate available to drive a cubic growth and a possible effect of the structure of iron stearate, the hydrated and dehydrated stearates have been here decomposed according to a previously published nanocube synthesis protocol [48], adapted from the Kovalenko method [28], and different ratios of NaOl and OA (keeping the same total molar amount of ligands, only the ratio is varied) were tested. The synthesis involved a quick heating (5 °C/min) to a step at 200 °C for 10 min, which is applied to favor the nucleation of NPs (improving the homogeneity of the mean size [31]) and then the reactant mixture is heated up to 315 °C with a heating rate of 1 °C/min and refluxed for 60 min under air. The same batch of iron precursors was used for the five tested ratios. Figure 1 showed TEM images of NPs obtained by tuning the type of iron stearates and the amount of NaOl. The mean size is determined from measurements of 300 NPs and is given with the standard deviation in Table 2, as well as the observed shape. Size distribution graphs are given in Figure S1.

As Figure 1 and Table 2 demonstrate clearly, the introduction of NaOl in the reactant media has an effect on the shape of NPs. The shape of the synthesized NPs being purely isotropic (spherical) only when sole OA is used as ligand. NaOl, even if added in low amount, triggers effectively the shape of NPs towards faceted shapes. However, the evolution of the shape with the amount of NaOl is not the same for all iron stearates and depends strongly on the nature of stearates, FeSt_2_ and FeSt_3_, and on their hydration degree.

Interestingly, specific shapes are preferentially obtained from a given precursor structure. At low NaOl/OA ratio (20/80), less rounded NPs are obtained for all precursors. When this ratio increases, FeSt_2_ and FeSt_2,d_ allowed to easily obtain nanoplates while that was not possible with FeSt_3_ and FeSt_3,d_ that led preferentially and quite easily to nanocubes. 

For a given NaOl/OA ratio, dehydrated precursors demonstrated to form sharper anisotropic morphologies compared to the hydrated precursors. Nonetheless, it was possible to improve the shape definition with hydrated precursors by increasing the part of NaOl in the synthesis. That is illustrated in Figure 1 with FeSt_3,d_ who led to cubes from a 50/50 NaOl/OA ratio while FeSt_3_ required a 100/0 ratio.

This study allows to conclude on two points for the shape control of IONPS. 

Firstly, hydration is actually a major parameter as hydrated precursors requested a larger amount of NaOl to tune the shape compared to their dehydrated version. This confirms our previous observation [45] that one parameter, which hampers the formation of well-defined anisotropic shapes is the lower availability of the oleate (Ol^–^) ligands, due to their interaction with water molecules. It is well-known that NaOl easily forms micelles with water [54], which would reduce the amount of NaOl that can interact with a nucleus to trigger the growth of given shape. That point explains the observation made with FeSt_3_ precursors and the ratio required to reach the cubic morphology.

Secondly, at a given ratio, precursors with a different structure (number of stearate chains) will lead to different shapes regardless of their hydration degree. Both FeSt_3_ precursors tend to form cubes whereas plates are observed for FeSt_2_.

The dependence of the shape from precursor nature requires at first to consider the theory behind formation of cubes and plates and involving the effect of the nature of the ligand. According to literature, the theory of the shape control through ligand absorption is quite simple. The crystallographic planes of fcc materials, such as magnetite, do not present the same surface energy γ. Typically, the ranking established from the lowest energy planes is $\gamma_{\left\{111\right\}}<\gamma_{\left\{100\right\}}<\gamma_{\left\{110\right\}}<\gamma_{\left\{\mathrm{hkl}\right\}}$ (with hkl > 1) [33,34]. When a nucleus is formed, it should be thus enclosed by the {111} planes that present the lowest surface energy. Thus, a cube which is enclosed by {100} planes would not be favored. But as for a given volume, the surface of a cube is lower than the one of an octahedron. The stable nucleus is thus a compromise with the presence of both planes families to reduce the surface energy: The cuboctahedron. Depending on the synthesis conditions, twin defects can also occur in the nucleus. The presence of defect in the nucleus will have a major effect on the final shape of the nanocrystal. For example, a mirror (111) plane can lead to flat nanoparticles [35]. The nucleus shape and structure appear thus major for the further shape control [55,56]. The role of the ligands in the shape control has been ascribed to the tuning of growth rate of certain planes of nuclei [23,28], e.g., the adsorption of a ligand on {111} planes of a cuboctahedron nucleus will reduce their growth rate. The {100} planes will grow faster leading to their disappearance. The final shape will then be an octahedron. Rath and al [36] showed through simulation that the {111} plane family of magnetite displays the most active surface for adsorption than the {311} and {110}. When a chelating ligand, such as oleate, is present, it would then adsorb on the {111} planes of the nuclei reducing its growth rate. 

Whereas it has been reported that the synthesis of cubes is explained by the selective adsorption of Ol^–^ ligand on nuclei controlling thus the cubic growth, the synthesis mechanism of iron oxide nanoplates is not clearly elucidated and their synthesis is still a challenge. Most reported nanoplates have been synthesized by hydrothermal process, but some nanoplates have been synthesized by thermal decomposition of iron oleate (FeOl) in presence of NaOl in octadecene [24,57,58]. By tuning the molar ratio of NaOl/FeOl in octadecene solvent, Zhou et al. [12] obtained Fe_3_O_4_{111} facet exposed nanoplates, truncated octahedrons, and tetrahedrons, and reported the predominant role of NaOl in preserving Fe_3_O_4_{111} facets [12]. The adsorption of Ol^–^ on the Fe_3_O_4_{111} facet is more favorable than that on the Fe_3_O_4_{100} facet. As a result, the growth on other facets is faster than that on the Fe_3_O_4_{111} facet, which results in the preservation of Fe_3_O_4_{111} facets in the products. Palchoudhury et al. [58] reported from a similar system that the formation of different-shaped NPs (including nanoplates) was achieved by controlling the nucleus concentration and growth rate and the nanoplates formation was attributed to the presence of a residual product from the precursor reaction and diffusional growth conditions.

In our case, the main difference between FeSt_2_ and FeSt_3_ are their water content and the amount of stearate chains, which is higher with FeSt_3_ than FeSt_2_. Moreover, we have reported previously [45] different decomposition kinetics with a decomposition of FeSt_2_ on a lower range of temperature than FeSt_3_ indicating that nucleation was favored with FeSt_2_ while growth was dominant for FeSt_3_. These two points could explain the role of the nature of the precursor on the NPs shape. In order to synthesize nanocubes, the growth step has to be controlled and sufficient amount of deprotonated carboxylic acid based/chelating ligands (i.e., oleate and/or stearate) need to be present in the reaction media to drive the growth. That is why FeSt_3_ precursor is optimal as it favors the nuclei growth over nucleation and provide more stearate chains and thus chelating ligands from its decomposition than FeSt_2_. In the case of the nanoplates, the control of the nucleation step seems to be central to get a flat nucleus. FeSt_2_ favoring the nucleation with its faster decomposition is then the most appropriate precursor. Although the nature of the precursor is major for the nanoplate shape production, the right ratio of ligands needs to be introduced. A precise ratio of binary surfactants have been reported for the formation of gold nanoplates [55] or iron oxide [58]. As oleic acid is adsorbed less strongly (OA is a universal stabilizer of each facet [12]) than deprotonated carboxylic acid/chelating ligands on specific faces of nuclei, their different adsorption on specific faces should also drive the growth towards the nanoplate shape.

The nanocubes and nanoplates are very interesting for biomedical applications, due to their shape anisotropy. Yet, the shapes obtained in this part could be optimized in order to get a properly defined shaped before investigating their structural properties. 

### 3.2. An Optimization of the Cubic Shape NPs Leading to Octopod Shape NPs

The optimization of the cubic shape of the NPs was realized in two steps. At first, the influence of the heating rate on the cubic morphology was investigated. Indeed, the kinetic conditions would favor the cubic shape [39]. Several reports [24,38,39,40] showed that high heating rates were rather used for nanocube synthesis. The heating rate has been varied by using hydrated FeSt_2_ (cheapest and easiest precursor to synthesize) at a NaOl/OA ratio of 50/50. Figure S2 presents the TEM images of syntheses, which have led to faceted NPs with no specific shapes with a heating rate of 1 °C/min and rounded corner cubes with a heating rate of 5 °C/min, confirming that a high heating rate is favorable to the cubic shape in our synthesis conditions. The increase of the heating rate led also to a decrease in the NPs mean size from 13.5 ± 2.5 nm for 1 °C/min to 11.8 ± 1.8 nm for 5 °C/min. 

**Figure 2:**
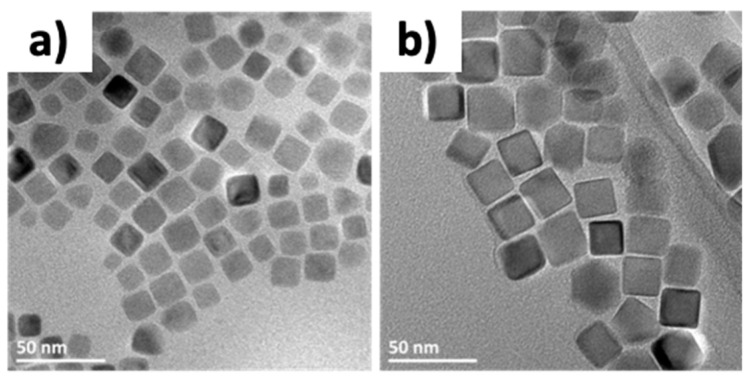
Transmission electron microscopy (TEM) images of nanocubes synthesized from FeSt_3_ using adapted Kovalenko protocol (**a**), and using the optimized protocol (**b**).

Secondly, the synthesis of nanocubes with FeSt_3_ conducted to rounded corner cubes by comparison with the oleate precursor that led to nanocubes with straight faces. The hypothesis being that the shape with round corners originated from a lack of the surfactant (NaOl) involved in the cubic growth control (in agreement with the presence of water molecules in the precursor). The objective was here to introduce more NaOl (i.e., increasing the ratio NaOl/FeSt_3_) to provide more ligands to control the growth of flat faces. Therefore, the ratio Precursor/Ligands was here increased from 0.75 to 1 just by adjusting the amount of FeSt_3_. Furthermore, a high heating ramp of 15 °C/min was chosen to ensure the growth step to happen in the kinetic regime, i.e., fast enough to favor the cubic morphology, which is not favored in the thermodynamic controlled conditions. TEM image in Figure 2 shows that such modification of the synthesis protocol allowed improving the definition of the nanocube shape and nanocubes with a mean size of 14.5 ± 1.6 nm (NC15) were obtained.

The XRD pattern of NC15 (Figure S3a) indicated that nanocubes are made of a core of wüstite Fe_1−x_O coated with a shell of spinel, as already observed [48,49]. Considering our previous results with nanospheres, such composition may be related to the competition between the growth rate and the oxidation kinetic of the Fe_1−x_O nuclei within this temperature range. Lattice parameters calculated for NC15 from Rietveld refinement gave a parameter of 8.392 ± 1 Å for the shell close to that of stoichiometric magnetite (0.8396 nm, JCPDS file 19-629) and of 4.237 ± 1 Å for the wüstite core. From an earlier study on nanocubes synthesized using iron oleate as precursor [48] and from recent results to obtain nanocubes or nanospheres with a homogeneous spinel composition [48,59], the first formed phase under the used experimental conditions should be the Fe_1−x_O one, which is then oxidized with time. The shell displayed generally a lattice parameter close or slightly larger to that of stoichiometric magnetite, which is ascribed to an epitaxial matching between both phases and strains, generated by the oxidation of wüstite and the lattice parameter of the wüstite phase strongly depended on its oxidation state. According to the formula Fe_1−x_O, the iron content *x* can be easily calculated from the cell parameter using the relationship *a*_FexO_ = 0.3856 + 0.0478*x* [60]. The result gives *x* value of 0.8, which suggest that the wüstite core has been formed in strong reducing conditions and even that the current synthesis conditions would be stronger than those used in our previous study (max 0.83) [48]. The wüstite phase was shown to be metastable and to transform into wüstite with higher iron content and spinel phases through an oxidation mechanism with ageing time in former nanocubes [48]. The oxidation mechanism and the epitaxial growth resulted in the diffusion of cations and vacancies that generated high strains at the Fe_1−x_O/Fe_3−x_O_4_ interface and in the Fe_3−x_O_4_ shell. Infra-red (IR) spectra (Figure S3) showed that the Fe–O bands are characteristic of a slightly oxidized magnetite. The broad band maximum is between those of the stoichiometric magnetite and maghemite (γ-Fe_2_O_3_) phases [31].

Different synthesis conditions have been tested in order to obtain nanocubes with a homogeneous composition in the spinel phase (i.e., without a wüstite core): Bubbling of air, other solvents than alkene; among others. Unfortunately, a loss of the cubic shape was generally observed. Indeed, the synthesis conditions of the cubic shaped NPs are constrained and these reducing and strict conditions should affect the oxidation kinetic. It has been often observed that such a combination of NaOl and OA leads to core-shell cubes, which suggested a strong influence of the amount of oleate chains in agreement with our previous observations during the synthesis of nanocubes using the iron oleate precursor [48]. 

**Figure 3:**
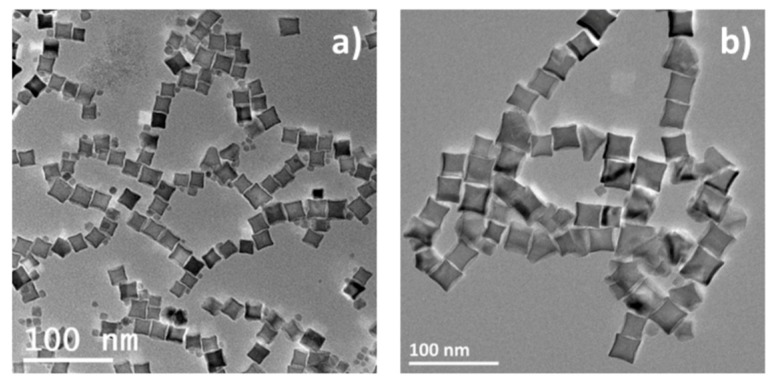
Transmission electron microscopy (TEM) images of the 18 nm octopods (**a**) and 28 nm octopods (**b**).

Nevertheless, other published methods have reported nanocubes with a spinel composition [20,21,39,43]. The method developed by Pellegrino and al [20] using a mixture of squalene and dibenzylether (DBE) as a solvent has led to nanocubes with a homogeneous composition. They reported that the products of decomposition of DBE were responsible for the control of the shape of NPs [21]. We adapted the Pellegrino team’s protocol using iron (III) stearate and DBE as solvent and it led to NPs with a cubic shape with elongated corners named octopods (Figure 3). Such octopods display interestingly a homogeneous spinel composition indicating that the reaction media was oxidizing by comparison with our one. 

Such an octopod shape would originate from a difference in the kinetics of the reaction. It has been explained that when the cubic NP is formed, if the number of monomers that precipitated on the surface is large enough, it will preferentially remain on the corners that present the higher surface energy instead of diffusing on the whole surface of the NPs. As the heating rate up to the growth step is rather fast (10 °C/min), this could be what is at work here. In Pellegrino’s protocol, the ratio DBE:squalene was demonstrated to drive size control. The more DBE was introduced, the smaller the NPs. 

In our synthesis conditions, the size has been tuned by adapting the FeSt:OA ratio and mean sizes of 17.2 ± 2.2 nm (NO18) for a ratio of 1:3 and of 27.8 ± 4.2 nm (NO28) for a ratio of 1:4.5 were thus synthesized. These objects are quite interesting as the presence of the elongated corner should increase the shape anisotropy of NPs. Their homogeneous spinel composition was confirmed by XRD refinement (Figure S3c) with a lattice parameter of 8.364 Å for NO18 and of 8.370 Å for NO28. Those lattice parameters values in-between those of magnetite and maghemite (0.8346 nm, JCPDS file 39-1346) indicated an oxidation of both NO. The position of the maximum of the broad Fe–O band (Figure S3d) between that at 580 cm^−1^ of magnetite and that at 630 cm^−1^ of maghemite is also in agreement with an oxidized magnetite composition. 

Due to the anisotropic shape, the crystallite size has been determined from Rietveld refinement as a function of the (hkl) planes (Table 3). The crystallites size depends on the size with the largest directions being <400> and <511> for NO28 and <220> and <440> for NO18. The longest directions for NO18 are thus the one belonging to the <110> family implying that the diagonal of the face is longer than the diagonal of the assimilated cube. For NO28, the longest direction is the <400> one followed by the <511> one which indicates a different exposition of crystallographic planes in the corner. For both size NO, the measured crystallite size matches with the size determined from TEM images with 15 ± 1 nm <220> for NO18 and 25 ± 1 nm <400> for NO28.

### 3.3. Exploring the Nanoplates Synthesis

Previously, all synthesis conditions led to the same plate thickness (around 6 nm). To tune this thickness in order to modulate the properties of the objects, the heating rate has been varied. With a NaOl/OA ratio of 80/20 and whatever the heating rate (Figure S4), nanoplates are obtained. The mean sizes are for 1 °C/min: l = 16.7 ± 5.2 nm and t = 8.6 ± 1.7 nm and for 5 °C/min: l = 16.6 ± 4.9 nm and t = 6.1 ± 1 nm): The thickness decreases with the increase of the heating rate. An increase in the heating rate up to 10 °C/min promoted partly the formation of cubic NPs (Figure S4). At 10 °C/min, the length decreased (15.8 ± 4.5 nm) and the thickness is in between the two former values (7.1 ± 1.4 nm). Yet, the variation is not significant in regard of the measurement error. Thus, there is no variation of the thickness with the heating rate.

**Figure 4:**
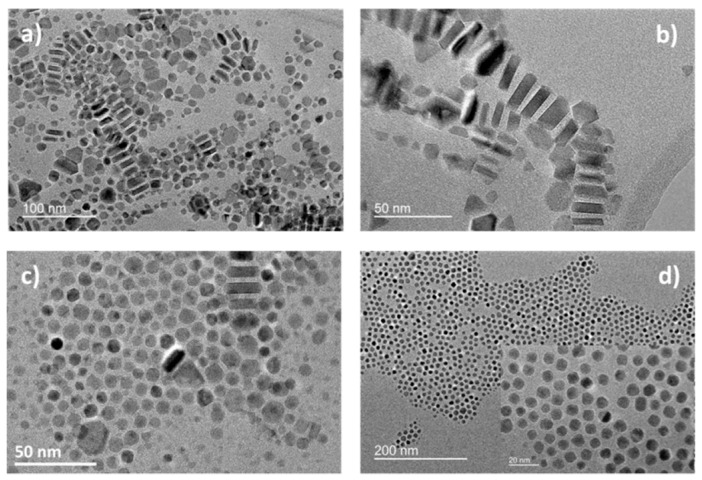
Influence of the germination step with the ratio 80/20: No germination step (**a**) germination step at (**b**) 190 °C for 10 min; (**c**) 210 °C for 10 min, and (**d**) 210 °C for 30 min.

As the nanoplates shape did not present the same dependence on the heating rate than the cubic one, it confirms that the main step to control the nanoplates shape is not the growth step (case of nanocubes), but rather the nucleation one. We have thus introduced a “nucleation step” in our experimental conditions of nanoplates synthesis and we have varied the “nucleation step” temperature. When this step was removed or was below 200 °C, nanoplates were observed to form (Figure 4). Above 200 °C, the nanoplate’s occurrence decreased. Moreover, when the duration of the step was increased from 10 to 30 min at 210 °C, no plates were observed at all (Figure 4). That confirms the importance of the nucleation step.

High resolution TEM (HR-TEM) and 3D TEM tomography have already been performed earlier on nanocubes to confirm their shape [48]. They were performed here on the nanoplates as this shape has been rarely reported. 

**Figure 5:**
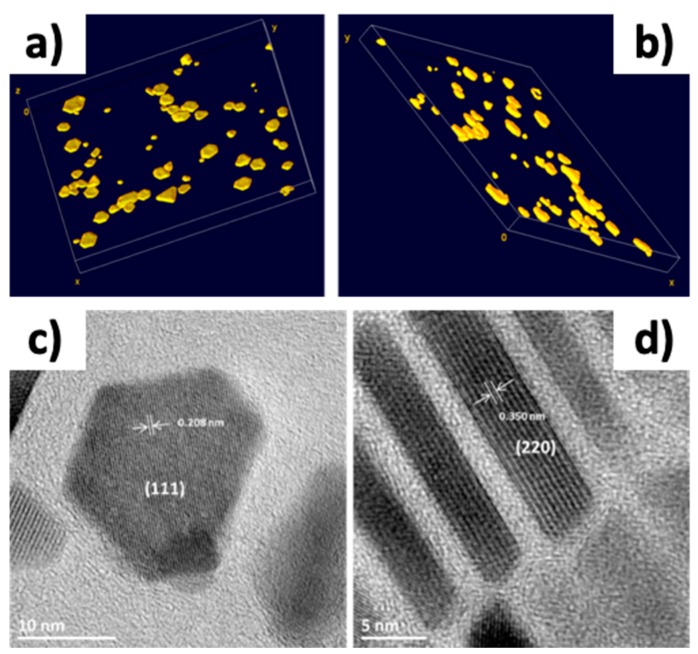
3D TEM reconstruction of the nanoplates (**a**,**b**) and high resolution transmission electron microscopy (HR-TEM) images of the long face of a platelets (**c**); and of the side (**d**).

The 3D TEM tomography images in Figure 5 confirmed the flat shape of nanoplates and showed that the fully formed nanoplates are not perfectly flat, but concave. The smaller NPs are rather convex confirming the spherical profile to reduce the surface energy. The plates studied here have a 17.5 ± 4.4 nm length and a 6.3 ± 1.3 nm thickness (nanoplates from hydrated FeSt_2_). HR-TEM images (Figure 5) showed that the long face of the plate is made out of {111} planes and the sides are made out of {220} planes.

The XRD pattern (Figure S3c) presented the XRD peaks characteristic of a spinel phase with a lattice parameter of 8.384 ± 1 Å, determined through Rietveld refinement. The nanoplates are thus slightly oxidized in agreement with the IR spectrum (Figure S3e) where a broad band with a maximum at 580 cm^−1^ (close to the magnetite one) is observed. The crystallite size according to specific crystallographic directions was also determined by Rietveld refinement (Table 3). The crystallite size of 17 ± 1 nm in the direction <111> is in agreement with the TEM size (16.7 ± 5.2 nm), but also with a 2D growth formation mechanism. As most of the nanoplates tended to lay flat during the XRD acquisition. It is hard to clearly see the planes within the thickness. Yet the value of 8 ± 1 nm close to the one measured for the thickness would imply that the planes (400), (511) enclose the sides of the plates.

We have shown above that high heating rates were required to favor the cubic shape when it was not the case for the nanoplate formation. Contrarily to the cubes, they do not present a reduced phase core of wüstite. However, plates whose thickness is very small should be easily oxidized when exposed to air despite the growth kinetics. 

## 4. Conclusions

The influence of the structure of the precursor on the shape of the NPs has been scarcely investigated in the literature. We have thus studied the thermal decomposition of hydrated and dehydrated FeSt_2_ and FeSt_3_ as a function of the NaOl/OA ratio introduced in the reaction media. We have mainly confirmed that (1) NaOl triggered effectively the NPs shape formation, that (2) the nature of the precursor affects the shape with FeSt_2_ favoring the formation of nanoplates whereas FeSt_3_ favored that of nanocubes and that (3) the hydration is deleterious for the formation of anisotropic shapes and more NaOl is needed to form anisotropic shaped NPs from highly hydrated precursors, most likely due to the interaction of ligands with water to form micelles. Thus, dehydrated precursors allowed a better control over the shape. The formation of nanocubes was confirmed to be driven by the amount of sodium oleate and the heating rate. The control of the monomer formation at the nucleation step was found very important for the nanoplate synthesis. Therefore, the nucleation step appeared very important to direct the nanoplate formation conjointly with a precise NaOl/OA ratio. Further work will focus on the study the magnetic properties of the obtained shapes.

## Figures and Tables

**Table 1:** Some reported nanocubes syntheses from literature.

Reference	Precursor	Solvent	Ligand	Reflux T and Duration	Heating Rate	Observations
[24]	Fe(acac)_3_	DBE	OA	290 °C/30 s	20 °C/min	Size and shape control with time and quantity of DBE
	Fe(acac)_3_	DBE	OA/4-Bisphenyl carboxilic acid	290 °C/30 s	20 °C/min	Smaller cubes
[9]	Fe(Ol)_3_	OD or TOA	OA	340 °C/4 h	10–15 °C/min	Size controled with T
[30]	Fe(acac)_3_	DBE	OA/4-Bisphenyl carboxilic acid	290 °C/30 s	ND	
[25,26]	Fe(Ol)_3_	Squalane	NaOl/OA	315 °C/2h	20 °C/min	Core-shell
[29]	Fe(acac)_3_	DBE	OA/HDD /OAm	290 °C/1h	15 °C/min	If heating rate increases along shorter reflux bigger NPs; on the opposite smaller NPs
[7]	Fe(Ol)_3_	eicosane	NaOl/OA	350 °C/30 s	3.3 °C/min	Core-shell
[46]	Fe(Ol)_3_ in situ	OD	NaOl	315 °C/ 2 h	ND	Shape control through amount of NaOl
[47]	Fe(Ol)_?_	OD	OA	320 °C/30 s	5.5 °C/min	
[12]	Fe(Ol)_3_	OD	NaOl/OA or DBAOL	315 °C/30’	3.3 °/min	
[27]	Fe(Ol)_3_	OD	NaOl /OA	315 °C/30 s	4 °C/min	Ratio Fe(Ol)/NaOl control the size
[28]	Fe(St)_2_	OA	NaOl /OA	380 °C/2 h	5 °C/min	
[23]	Fe(acac)_3_	Squalane	Decanoic acid/DBE	310 °C/1 h	7 °C /min	Size controled with ratio squalane/DBE

DBE: Dibenzylether; OA: Oleic acid; TOA: Trioctylamine; HDD: Hexadecylamine; OAm: Oleyamine; OD: Octadecene; DBAOL: Dibutyammonium oleate; ND: Non Disclosed.

**Table 2:** Size and shape of nanoparticles as a function of the nature and amount of reactants (M: Majority, m: minatory, r: rare).

Precursor		0/100	20/80	50/50	80/20	100/0
FeSt_2_	Size (nm)	9.4 ± 1.9	11.7 ± 2.5	13.5 ± 2.5	17.5 ± 4.4 (L) 6.3 ± 1.3 (t)	7.2 ± 2.4
	Shape	Spheres	Quasi octahedron	Deformed Cubes	Plates	Faceted
FeSt_2,d_	Size (nm)	10.0 ± 1.5	10.9 ± 1.4	10 ± 0.6	13.5 ± 5.2 (L) 66.5 ± 1.7 (t)	9 ± 2.7
	Shape	Spheres	Quasi spherical	Cubes	Plates	Various faceted plates
FeSt_3_	Size (nm)	10.8 ± 1.7	12.4 ± 1.7	8.3 ± 2.2	11.3 ± 4.3	11.7 ± 1.5
	Shape	Spheres	Quasi spherical	Faceted	Faceted	Cubes
FeSt_3,d_	Size (nm)	9.2 ± 1.5	9.8 ± 1.4	15.3 ± 1.8	20 ± 1.9/14 ± 1.4	13.9 ± 2.2
	Shape	Spheres	Quasi spherical (M)/Octahedrons©	Cubes	Cubes (M)/Plates (m)	Elongated cubes

For plates: L for length and t for thickness.

**Table 3:** TEM size and lattice parameter and crystallites size determined from X-ray diffraction (XRD) refinement.

Sample	TEM (nm)	Lattice Parameter (Å)	Crystallite Size According to Crystallographic Direction ± 1 (nm)										
			220	311	222	400	331	422	511	333	440	531	442
NO18	17.2 ± 2.2	8.364	15	13	11	11	11	12	13	11	15	12	11
NO28	27.8 ± 4.2	8.370	19	20	13	25	18	17	23	12	19	21	16
NPl17	16.7 ± 5.2	8.384	11	9	17	8	11	10	8	17	11	9	12

## Supplementary Materials

The following are available online at http://www.mdpi.com/2079-4991/8/11/881/s1, Figure S1: Size distribution of NPs determined from TEM diameter measurement over 300 NPS, Figure S2: TEM images of the decomposition of hydrated FeSt_2_ at a NaOl/OA ratio of 50/50 with a heating rate of (a) 1 °C/min and (b) 5 °C/min, Figure S3: XRD refinement of NC15 (a), NPl (b), NO28 (c), IR spectra of NC (blue), NPl (yellow) and NO28 (green) (d) and typical IR spectra of slightly oxidized magnetite and maghemite (e), Figure S4: TEM images of the decomposition of hydrated FeSt_2_ at a NaOl/OA Ratio 80/20 (a) 1 °C/min, (b) 5 °C/min and (c) 10 °C/min.
