# Supplementary material for: Unravelling the Thermal Decomposition Parameters for The Synthesis of Anisotropic Iron Oxide Nanoparticles
Source: Nanomaterials (Basel). 2018 Oct 29;8(11):881. doi: 10.3390/nano8110881 (PMC6266703; doi:10.3390/nano8110881)
Supplement: Supplementary file 1 [file nanomaterials-08-00881-s001.pdf]

Supplementary Information

Unravelling the Thermal Decomposition Parameters for the Synthesis of Anisotropic Iron Oxide Nanoparticles

Geoffrey Cotin [1,2], Céline Kiefer [1,2], Francis Perton [1,2], Dris Ihiawakrim [1,2], Cristina Blanco-Andujar [1,2], Simona Moldovan [1,2], Christophe Lefevre [1,2], Ovidiu Ersen [1,2], Benoit Pichon [1,2], Damien Mertz [1,2] and Sylvie Bégin-Colin [1,2,*]

1 Institut de Physique et Chimie des Matériaux de Strasbourg, UMR 7504, University of Strasbourg, CNRS, F-67034 Strasbourg, France; geoffrey.cotin@ipcms.unistra.fr (G.C.); celine.kiefer@ipcms.unistra.fr (C.K.); francis.perton@ipcms.unistra.fr (F.P.); dris.ihiawakrim@ipcms.unistra.fr (D.I.); cristina.blancoandujar.09@ucl.ac.uk (C.B.A.); simona.moldovan@ipcms.unistra.fr (S.M.); Christophe.lefevre@ipcms.unistra.fr (C.L.); ovidiu.ersen@ipcms.unistra.fr (O.E.); benoit.pichon@ipcms.unistra.fr (B.P.); damien.mertz@ipcms.unistra.fr (D.M.)

2 Labex CSC, Fundation IcFRC/University of Strasbourg, 8 allée Gaspard Monge BP 70028 F - 67083 Strasbourg Cedex.

* Correspondence: sylvie.begin@ipcms.unistra.fr; Tel.: +33-388-107-192

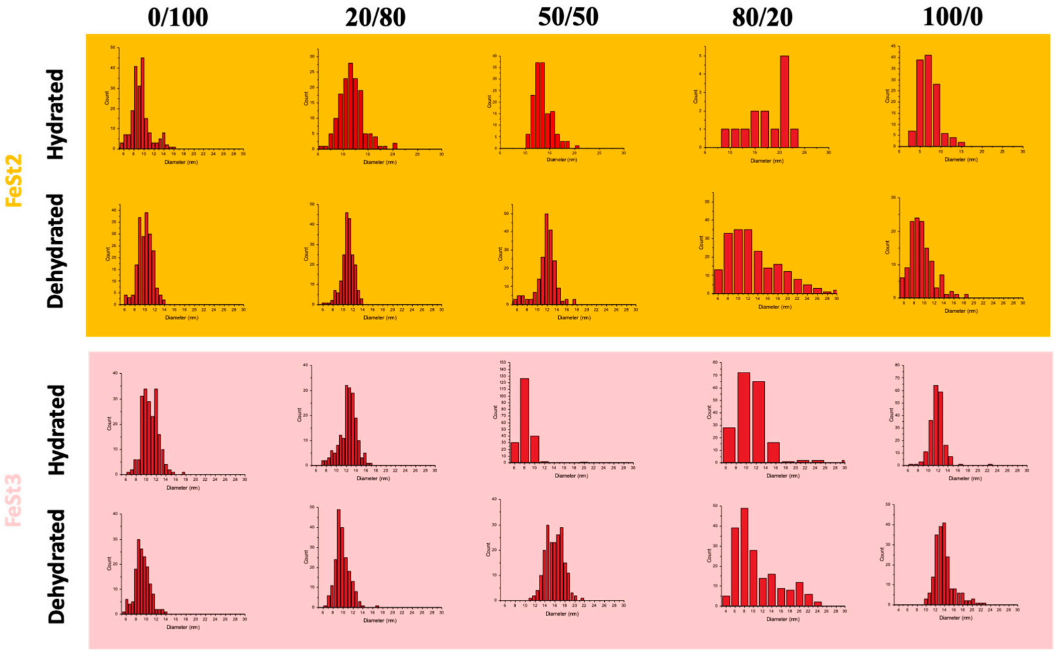

Figure S1. Size distribution of NPs determined from TEM diameter measurement over 300 NPS.

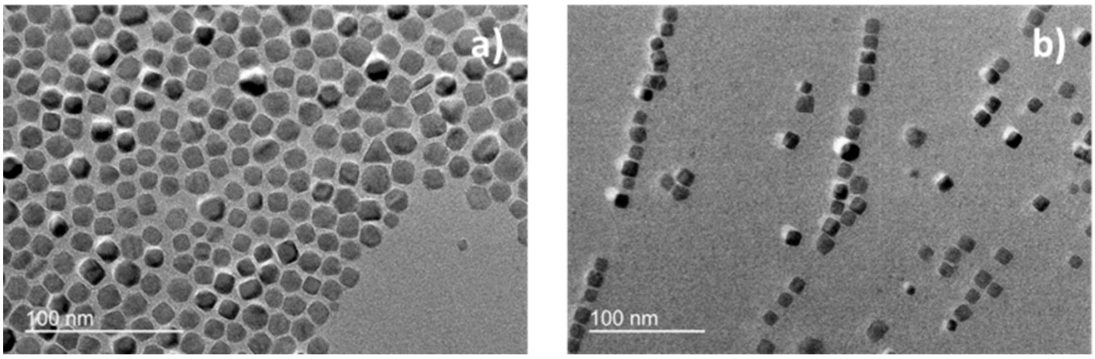

Figure S2. TEM images of the decomposition of hydrated $FeSt_2$ at a NaOl/OA ratio of 50/50 with a heating rate of (a) 1 °C/min and (b) 5 °C/min.

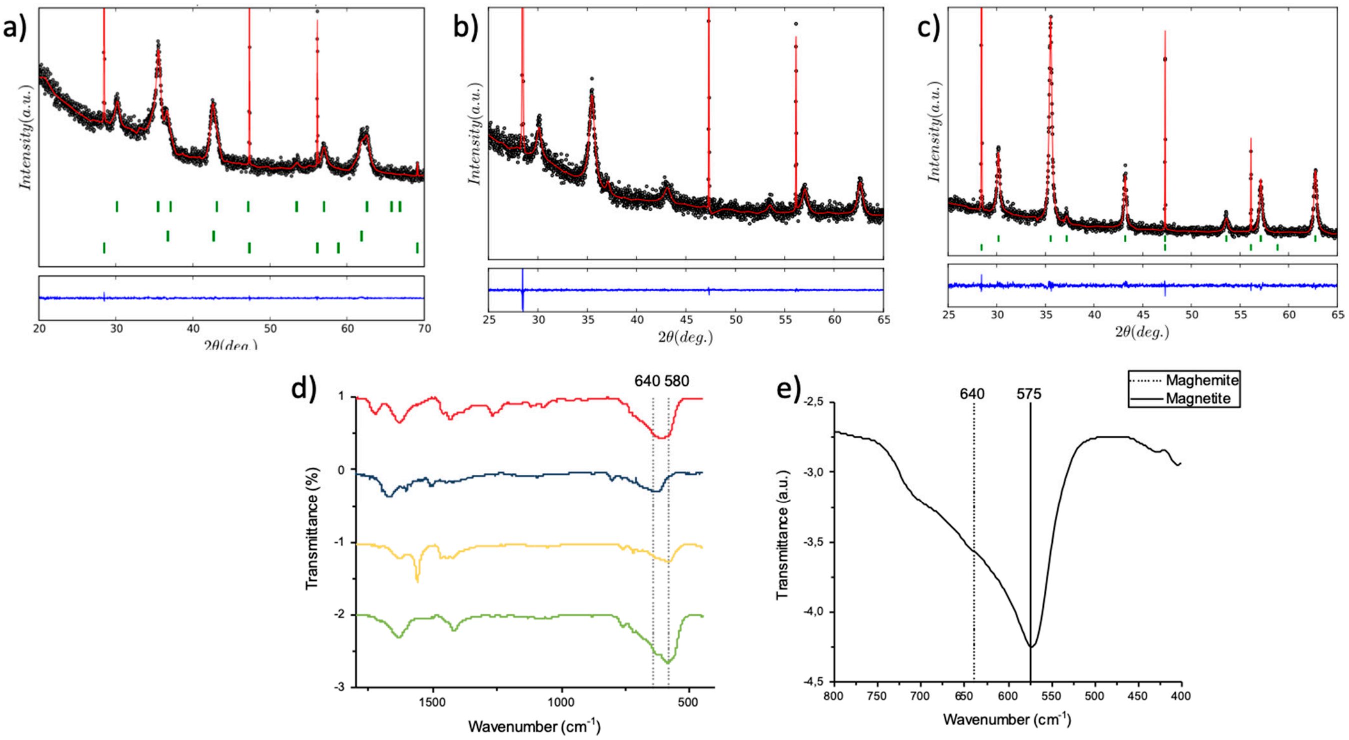

Figure S3. XRD refinement of NC15 (a), NPl (b), NO28 (c), IR spectra of NC (blue), NPl (yellow) and NO28 (green) (d) and typical IR spectra of slightly oxidized magnetite and maghemite (e).

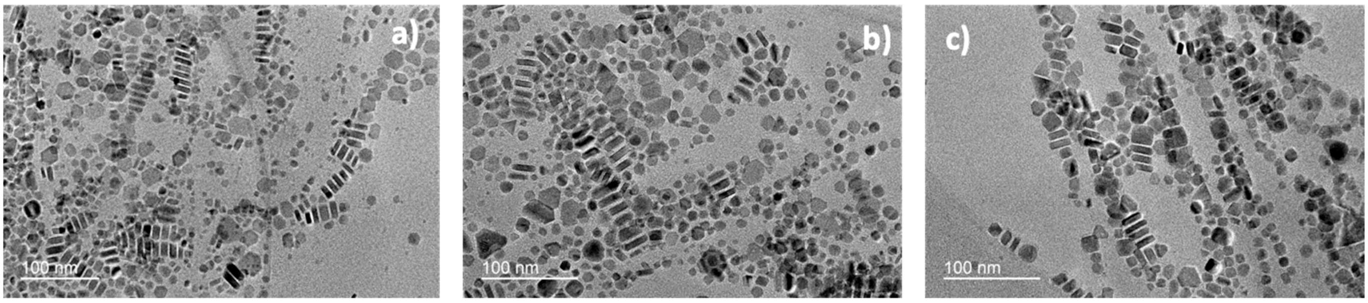

Figure S4. TEM images of the decomposition of hydrated $FeSt_2$ at a NaOl/OA Ratio 80/20 (a) 1 °C/min, (b) 5 °C/min and (c) 10 °C/min.
